# Associations Between Occupational Noise Exposure, Aging, and Gender and Hearing Loss: A Cross-Sectional Study in China

**DOI:** 10.3390/audiolres15040091

**Published:** 2025-07-23

**Authors:** Yixiao Wang, Peng Mei, Yunfei Zhao, Jie Lu, Hongbing Zhang, Zhi Zhang, Yuan Zhao, Baoli Zhu, Boshen Wang

**Affiliations:** 1 School of Public Health, Nanjing Medical University, Nanjing 211166, China; yixiaowang@stu.njmu.edu.cn (Y.W.); yunfeizhao@stu.njmu.edu.cn (Y.Z.); 2 Jiangsu Provincial Center for Disease Prevention and Control (Jiangsu Provincial Academy of Preventive Medicine), Nanjing 210009, China; njmumeipeng@163.com (P.M.); eye00@126.com (H.Z.); zhangzhi@jscdc.cn (Z.Z.); yuanz@jscdc.cn (Y.Z.); 3 Party and Government Office, Community Health Management Center, Kunshan 215300, China; 4 Key Laboratory of Environmental Medicine Engineering of Ministry of Education, Southeast University, Nanjing 210009, China; 220233996@seu.edu.cn; 5 Jiangsu Preventive Medical Association, Nanjing 210009, China

**Keywords:** occupational noise exposure, hearing loss, age-related hearing loss, gender, machine learning

## Abstract

**Background:** Hearing loss is increasingly prevalent and poses a significant public health concern. While both aging and occupational noise exposure are recognized contributors, their interactive effects and gender-specific patterns remain underexplored. **Methods:** This cross-sectional study analyzed data from 135,251 employees in Jiangsu Province, China. Demographic information, noise exposure metrics, and hearing thresholds were obtained through field measurements, questionnaires, and audiometric testing. Multivariate logistic regression, restricted cubic spline modeling, and interaction analyses were conducted. Machine learning models were employed to assess feature importance. **Results:** A nonlinear relationship between age and high-frequency hearing loss (HFHL) was identified, with a critical inflection point at 37.8 years. Noise exposure significantly amplified HFHL risk, particularly in older adults (OR = 2.564; 95% CI: 2.456–2.677, *p* < 0.001), with consistent findings across genders. Men exhibited greater susceptibility at high frequencies, even after adjusting for age and co-exposures. Aging and noise exposure have a joint association with hearing loss (OR = 2.564; 95% CI: 2.456–2.677, *p* < 0.001) and an interactive association (additive interaction: RERI = 2.075, AP = 0.502, SI = 2.967; multiplicative interaction: OR = 1.265; 95% CI: 1.176–1.36, *p* < 0.001). And machine learning also confirmed age, gender, and noise exposure as key predictors. **Conclusions:** Aging and occupational noise exert synergistic effects on auditory decline, with distinct gender disparities. These findings highlight the need for integrated, demographically tailored occupational health strategies. Machine learning approaches further validate key risk factors and support targeted screening for hearing loss prevention.

## 1. Introduction

Noise-induced hearing loss (NIHL) is the long-term cumulative exposure to high noise levels that impairs the auditory system and causes sensorineural hearing loss, usually bilateral [1,2]. Occupational hearing loss is a worldwide health problem [3,4]. The World Health Organization (WHO) estimates that around 1.57 billion individuals globally have experienced hearing loss since 2019 [5]. Recently, noise-induced deafness (NID) related to occupational exposure has emerged as the second most prevalent occupational illness in China, following pneumoconiosis [6]. Over 10 million employees in China are presently subjected to detrimental noise levels [7]. Studies showed that almost all adults experience bilateral hearing loss in later life, including progressive and irreversible bilateral hearing loss [8]. Hearing impairment may result in a range of issues, including diminished engagement in social activities and the workforce, a heightened likelihood of experiencing social isolation, and declining mental well-being [9,10,11].

Everyone’s hearing declines to some extent as they age. According to national health surveys conducted in the United States from 2000 to 2008, hearing loss is more common among older adults, accounting for 45% of those aged 60 to 69 years and 68% of those aged 70 to 79 years [12]. As a global standard, ISO 1999 [13] demonstrates the progressive escalation of auditory thresholds within the frequency spectrum of 3–8 kHz. However, studies have shown that the loss of outer hair cells is usually much smaller in older people who live in quiet environments [14]. The effects of noise or ototoxins on age-related hearing loss (ARHL) are thought to be cumulative, resulting in the substantial loss of outer hair cells [15,16].

Relevant experimental data suggest that early noise exposure might damage inner hair cell synapses [17]. In one experiment in rats, older rats exposed to noise experienced more significant loss of the mid-basal IHC-ANF synapses than younger rats exposed to noise [18]. Human temporal bone studies have shown that noise affects ARHL, with more pronounced hearing impairment occurring at elevated frequencies [19].

Moreover, recent studies have highlighted gender-specific vulnerabilities in the development of NIHL. An epidemiological analysis report showed that, compared to women, men have a significantly higher prevalence of high-frequency hearing loss, even in low-exposure environments [20]. Biological differences, particularly hormonal influences such as estrogen, are believed to confer auditory protection in females [21,22]. On the contrary, men often have higher occupational noise exposure levels and exhibit earlier signs of hearing deterioration [23]. However, some researchers argue that lifestyle, occupational segregation, and use of hearing protection may confound observed gender disparities [9,24,25]. Despite ongoing debate, accumulating mechanistic and population-based evidence has indicated that gender is a key modifier in ARHL and NIHL susceptibility. Understanding these differences is essential for tailoring occupational health interventions to protect vulnerable subpopulations.

By analyzing noise exposure data from employees of relevant enterprises in Jiangsu Province, China, this study aimed to gain a comprehensive understanding of the impact of noise exposure on hearing loss across different age groups and genders. The findings are expected to enhance the understanding of the health consequences of workplace noise exposure and contribute valuable evidence to the field of occupational health, which will inform the development of more effective occupational health and safety policies to ensure that employees have a healthier and safer hearing environment at work.

## 2. Methods

### 2.1. Study Population

The study population consisted of 135,251 employees who were monitored by the Jiangsu Provincial Center for Disease Control and Prevention in 2021. Subjects’ general information, including gender, age, medical history, history of occupational exposure, and usage of hearing conservation measures, was collected through an occupational health questionnaire. Data on enterprise working environment monitoring and occupational health examinations were obtained from the Jiangsu Province Occupational Disease Surveillance System, which has been approved for use by the Jiangsu Provincial Center for Disease Control and Prevention.

The inclusion criteria were as follows: (a) long-term employment contract and (b) confirmation of signing the informed consent form. And the criteria for exclusion included the following: (a) a familial history of hereditary hearing loss or a personal history of conditions such as deformities of the ear canal, sudden hearing loss, or perforation of the tympanic membrane; (b) a history of diseases affecting the auditory system (otitis media, etc.) or a history of using ototoxic medications; and (c) incomplete information on occupational health monitoring.

Based on the findings from electrical audiometry during the occupational health assessment and the aforementioned inclusion and exclusion criteria, participants were categorized into two groups: a noise group and a control group, in accordance with the Emission Standard for Industrial Enterprises Noise at Boundary (GB 12348-2008) [26]. The criteria for classifying the noise group included the following: (a) exposure to occupational noise; and (b) an eight-hour equivalent continuous A-weighted sound pressure level (LAeq, 8 h) of 80 dB or greater. Subjects in the control group had no occupational noise exposure.

### 2.2. Pure-Tone Audiometry and NIHL Definition

Pure-tone audiometry (PTA) was conducted in a standardized soundproof room meeting the GB/T 16296.1-2018 [27] “Acoustics—Audiometric Test Methods” specifications. All participants refrained from occupational noise exposure for at least 24 h prior to testing to minimize the influence of temporary threshold shifts. According to the diagnostic criteria (GBZ 49–2014) [28], audiometric thresholds for both ears were measured at 0.5, 1, 2, 3, 4, and 6 kHz using the ascending method of pure-tone presentation. This frequency range is highly sensitive to cumulative noise exposure and early cochlear damage. When the unmasked air-conduction threshold in the test ear differed by ≥40 dB from the contralateral ear, masking procedures were applied to ensure accuracy. Testing was performed by trained audiology personnel using calibrated equipment in a controlled acoustic environment. In the present study, workers who had a binaural high-frequency threshold average (BHFTA) across 3, 4, and 6 kHz greater than 25 dB were classified as having NIHL, whereas those with BHFTA ≤ 25 dB were considered to have normal hearing [28,29]. Correspondingly, in this study, bilateral low-frequency average hearing was calculated based on the thresholds of participants at 0.5, 1, and 2 kHz.

### 2.3. Field Occupational Noise Level Detection

Occupational noise levels were measured according to the national standard Measurement of Physical Factors in the Workplace Part 8: Noise (GBZ/T 189.8–2007) [30] using Quest Noise Pro DL multifunctional personal noise dosimeters (Quest Technologies, Oconomowoc, WI, USA). Each dosimeter was field-calibrated using a Quest QC-10 acoustic calibrator before and after each use, following OSHA 29 CFR 1910.95 [31], ensuring accuracy within ±0.5 dB. Calibration procedures were logged, and devices outside the accepted range were recalibrated or replaced.

Sampling procedures adhered to GBZ/T 189.8–2007 [30]: When fewer than 3 workers were present at a given workstation, all were measured. For 3–5 workers, 2 were randomly selected; for 6–10, 3 were selected; and for more than 10, 4 workers were included. To enhance representativeness and risk assessment, at least one participant with the longest exposure duration and highest task-related noise intensity was deliberately included per group. Random selection was conducted using a computerized random number generator to ensure unbiased inclusion of other subjects.

Measurements were conducted during routine 8 h shifts under normal operating conditions. Workers were guided to wear the dosimeter properly, and data were recorded as time-weighted average A-weighted equivalent continuous sound levels (TWA, Leq dBA), reflecting real-world exposure intensity.

### 2.4. Machine Learning Models

In this study, machine learning techniques were employed to improve the identification and quantification of risk factors contributing to NIHL among individuals exposed to occupational noise. Three machine learning algorithms were selected for modeling based on complementary advantages and applicability to the characteristics of the dataset: Random Forest Classifier (RFC), Naive Bayes Classifier (NBC), and CatBoost Classifier.

RFC is a non-parametric ensemble learning algorithm based on decision tree bagging. It is robust to overfitting and handles mixed-type features well, making it suitable for datasets with both categorical and continuous variables [32,33]. NBC is a classification algorithm that is both simple and effective, and it is based on Bayes’ theorem. NBC is computationally efficient and performs well on linearly separable data with probabilistic structures, especially when features are conditionally independent [34,35]. CatBoost, a gradient boosting decision tree algorithm developed specifically for categorical data, automatically handles categorical feature encoding internally using ordered boosting and target statistics. It is highly suitable for real-world datasets with imbalanced class distributions and exhibits excellent generalization with minimal tuning [36,37,38]. The inclusion of these three diverse models enables a comparative evaluation across tree-based, probabilistic, and boosting paradigms.

All models utilized the same input features. Feature selection was embedded within the internal mechanisms of each classifier, and feature importance was computed using model-specific approaches. For the RFC, feature importance was calculated based on Gini impurity reduction (also known as Mean Decrease in Impurity), which quantifies the contribution of each feature to reducing class impurity when used to split decision nodes [39]. For the NBC, feature relevance was assessed through log-likelihood estimation under the assumption of a Gaussian distribution for continuous features, with importance inferred from their influence on class-conditional probability distributions [40]. For the CatBoost Classifier, the Prediction Value Change method was applied, which measures the change in prediction score when a given feature is permuted or removed, thereby capturing the marginal impact of that feature on model output [40].

For model evaluation, accuracy measures the overall correctness of predictions. Precision is the proportion of true positives among all instances predicted as positive, reflecting the model’s control over false positives. Recall is the proportion of true positives among all actual positive instances, reflecting the model’s ability to detect positives and avoid false negatives [41]. Together, these metrics provide a comprehensive assessment of classification performance and help ensure the model’s reliability and generalizability.

### 2.5. Covariates

The collection of demographic and occupational covariates was conducted through a series of structured interviews, physical examinations, and on-site sampling. It has been reported that manganese, a neurotoxic metal, can accumulate in brain regions involved in auditory processing and has been associated with elevated hearing thresholds and cochlear dysfunction [42,43]. Dust and high-temperature exposure are pervasive in the work environment of the study population and can exacerbate oxidative stress and systemic inflammation, indirectly impacting auditory health [43]. In our study, manganese, dust, and high temperature exposures were defined based on self-reported histories and, when available, validated against occupational monitoring records to reduce misclassification bias.

### 2.6. Data Analysis

Descriptive statistics were computed to summarize the characteristics of the study population. Data preprocessing included normality assessment using the Shapiro–Wilk test. Continuous variables were reported as the mean ± standard deviation (SD) or median with interquartile ranges (Q1 and Q3), depending on their distribution, while categorical variables were summarized using frequencies and percentages. Group comparisons between noise-exposed and control participants were conducted using independent-sample *t*-tests or Mann–Whitney U tests for continuous variables and chi-square tests for categorical variables.

To assess the impact of occupational noise exposure across the lifespan, we first evaluated the prevalence of noise-induced hearing loss (NIHL) in both the noise-exposed and control groups. Participants were stratified into five age categories (16–30, 31–40, 41–50, 51–60, and 61–76 years), and group-wise comparisons were conducted using chi-square tests to determine statistically significant differences in NIHL prevalence across age strata. To further investigate age-related trends in auditory function within the noise-exposed population, we calculated median hearing thresholds at six key audiometric frequencies (0.5, 1, 2, 3, 4, and 6 kHz), stratified by age group. This allowed us to characterize the progression of hearing loss across frequencies and life stages. In addition, we explored gender-related differences in hearing thresholds within both the noise-exposed and control groups. For each frequency, the median and 95th percentile thresholds were calculated separately for males and females. To adjust for the potential confounding effect of age, we employed analysis of covariance, with age included as a continuous covariate. This approach enabled us to isolate the independent effect of gender on hearing thresholds while accounting for age-related variation in auditory function.

Subsequently, multivariate logistic regression analyses were conducted to identify risk factors associated with HFHL. The model included age, gender, occupational noise exposure, and co-exposures to manganese, dust, and high-temperature environments as covariates. The results are presented as odds ratios (ORs) with corresponding 95% confidence intervals. To evaluate potential gender-specific effects, stratified logistic regression models were conducted separately for male and female subgroups.

To investigate the potential nonlinear association between age and the risk of HFHL, we applied a restricted cubic spline (RCS) regression model with age as a continuous exposure variable. Four knots were placed at the 5th, 35th, 65th, and 95th percentiles of the age distribution, allowing for a flexible yet parsimonious estimation of the dose–response relationship while minimizing the risk of model overfitting. This spline-based approach facilitated the detection of threshold effects and inflection points in the relationship between age and HFHL risk.

To evaluate the combined effect of occupational noise exposure and age on the risk of HFHL, joint and interaction analyses were performed using a dichotomized age variable, defined based on the inflection point derived from the restricted cubic spline model. In the joint analysis, participants were classified into four exposure categories according to age group (≤cutoff vs. >cutoff) and noise exposure status (yes/no). Multivariable logistic regression models were used to estimate adjusted odds ratios (ORs) and 95% confidence intervals (CIs) for each subgroup. All models were adjusted for potential confounders, including gender, and co-exposures to manganese, dust, and high-temperature environments. Interaction effects between age and noise exposure were assessed under both additive and multiplicative frameworks. For additive interaction, the Relative Excess Risk due to Interaction (RERI), Attributable Proportion (AP), and Synergy Index (SI) were calculated, with 95% confidence intervals estimated using the delta method. A positive RERI or an AP > 0, or an SI > 1, indicated a supra-additive interaction. For multiplicative interaction, a cross-product term (age × noise exposure) was introduced into the logistic regression model, and the statistical significance of the interaction term was evaluated. All interaction models were adjusted for the same set of confounders as in the main regression analysis.

In this study, machine learning techniques were employed to enhance the identification and quantification of risk factors contributing to NIHL among individuals exposed to occupational noise. For the machine learning analysis, all features were preprocessed and encoded prior to model training to ensure compatibility with the algorithmic input requirements. The binary classification target variable, HFHL, was encoded as 1 for presence and 0 for absence. Similarly, occupational noise exposure was treated as a binary variable, coded as 1 for exposed and 0 for unexposed individuals. The categorical variable gender was coded as 1 for male and 2 for female. Age was expressed as a categorical variable divided into five age groups, coded as follows: 1 for 16–30 years, 2 for 31–40 years, 3 for 41–50 years, 4 for 51–60 years, and 5 for 61–76 years. Noise exposure duration was expressed as a continuous variable over the years. We developed machine learning models using RFC, NBC, and CatBoost Classifier. The input variables for the three machine learning methods were as follows: age categories, gender, exposure time seniority, and exposure factor. Data were randomly split into training and test sets in a 70:30 ratio. Hyperparameters were optimized through grid search and 10-fold cross-validation. Ten-fold cross-validation was employed to reduce variance in the estimation of model performance compared to a single train–test split. The dataset was partitioned into k = 10 subsets (folds), with nine folds used to train the model and the remaining fold used to validate accuracy. This process was repeated k = 10 times, each time leaving out one fold as the validation set. The final model performance metrics were obtained by averaging the results across the ten validation folds. Finally, the model developed via ten-fold cross-validation was evaluated on the test sample. Model performance was evaluated using accuracy, recall, and precision metrics across training, cross-validation, and test datasets.

Gender, age category, exposure tenure, and exposure factor were used as inputs to Random Forest (RFC), Naive Bayes (NBC), and CatBoost models. Hyperparameter optimization was carried out via grid search over predefined ranges (e.g., number of trees, tree depth, learning rate) embedded within ten-fold cross-validation, with the combination yielding the highest average validation accuracy selected for each model. For RFC, the number of trees was set to 100, the minimum samples required to split an internal node was set to 2, the minimum samples required at a leaf node was set to 1, the maximum tree depth was set to 10, and the maximum number of leaf nodes was set to 50. CatBoost was configured with 100 iterations, a learning rate of 0.1, a maximum depth of 10, and early stopping after 20 rounds. NBC used its default Gaussian smoothing (α = 1). Data were shuffled before each fold to mitigate overfitting and ensure robust, generalizable results.

EpiData version 3.1 was used to enter and manage data. To ensure data integrity and minimize input errors, double data entry and cross-checking procedures were implemented. All statistical analyses were conducted using R version 4.4.3. Statistical significance was determined at a two-sided *p*-value threshold of <0.05. However, no corrections for multiple comparisons were made.

## 3. Result

### 3.1. General Information

This study involved a total of 135,251 participants, of whom 110,810 were in the noise-exposed group and 24,441 were in the control group, with an age range of 16–76 years. Noise-exposed participants were older on average (40.3 ± 10.4 years) than those unexposed (32.3 ± 8.8 years). Males predominated in both cohorts, and the proportion was higher in the noise-exposed group (74.1% vs. 69.1%), reflecting the gendered distribution of industrial labor sectors in China. The BHFTA in the noise exposure group was (24.1 ± 12.2 dB), which was higher than that in the control group (23.4 ± 8.8 dB) (*p* < 0.01). Environmental co-exposures were markedly more common among noise-exposed workers, particularly dust (49.8%) and high-temperature environments (8.8%), which were nearly absent in the control group. These disparities underscore the occupational complexity of the noise group and the importance of considering multiple risk factors in evaluating auditory outcomes (Table 1).

**Figure 1:**
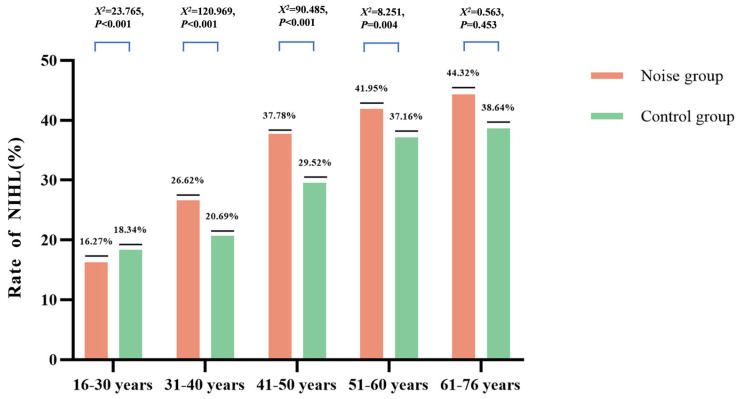
Prevalence of noise-induced hearing loss (NIHL) by occupational noise and age category.

As shown in Table 2 and Figure 1, the prevalence of NIHL increased progressively with age in both groups. However, except for the youngest group (16–30 years), NIHL was consistently more frequent in the noise-exposed participants. The most marked group difference appeared in the 41–50 age category, where the NIHL rate in the noise group exceeded that of the control group by 8.26%. Statistical analysis using the chi-square test revealed significant differences in NIHL prevalence across age groups, with the exception of the 61–76-year cohort (*p* < 0.05).

### 3.2. Analysis Results of Related Factors

**Figure 2:**
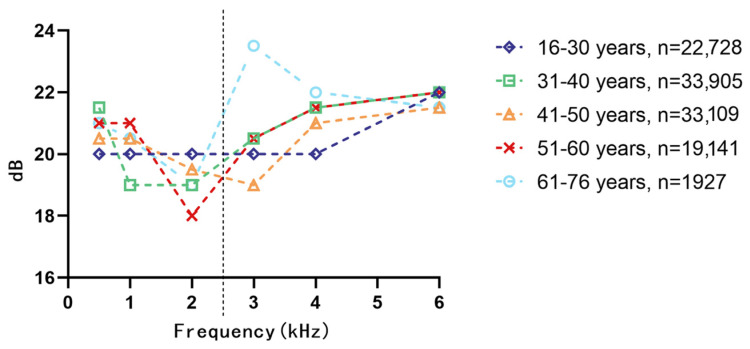
Median hearing thresholds across hearing thresholds stratified by age groups in the noise group.

Figure 2 presents the distribution of median hearing thresholds across different frequency bands, stratified by age in the noise-exposed group. In the low-frequency range (<2 kHz), thresholds remained relatively stable across age groups. However, in the high-frequency range (>2 kHz), median thresholds progressively increased with frequency, reflecting a typical pattern of age-related and noise-induced hearing loss. This trend was more pronounced among older individuals, who consistently exhibited higher thresholds at frequencies above 2 kHz, suggesting greater susceptibility to high-frequency auditory decline.

**Figure 3:**
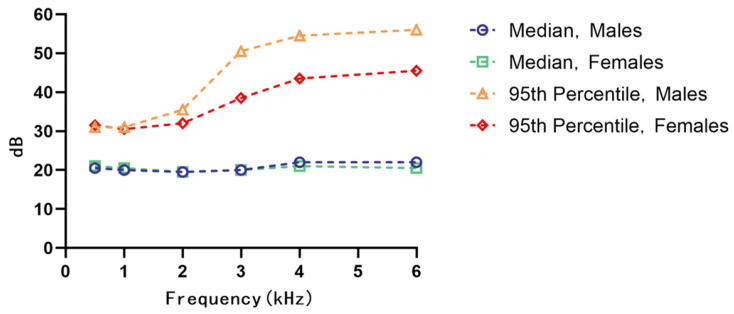
Median and 95th percentile values for hearing thresholds for the noise group categorized by gender.

To investigate the effect of gender on hearing thresholds within the noise-exposed group, both median and 95th percentile values were compared between males and females (Figure 3). At 2 kHz, median thresholds were similar for both genders. However, starting from 3 kHz, males consistently exhibited higher median thresholds than females, indicating poorer hearing sensitivity. The differences were more pronounced in the 95th percentile distribution, particularly at high frequencies, suggesting greater susceptibility to severe hearing loss in a subset of male participants. In order to control for the potential confounding effect of age, analysis of covariance with age as a covariate was employed, and this showed that the frequency difference between the male and female groups was statistically significant (*p* < 0.001). This confirms that the observed differences were not entirely attributable to age-related changes.

In the control group, gender-related differences in median thresholds were less consistent. However, 95th percentile values remained higher among males, especially in the high-frequency range (Table 3), indicating a greater risk of extreme hearing threshold elevations among male participants, even in the absence of occupational noise exposure.

### 3.3. Multivariate Logistic Regression Analysis Results

**Figure 4:**
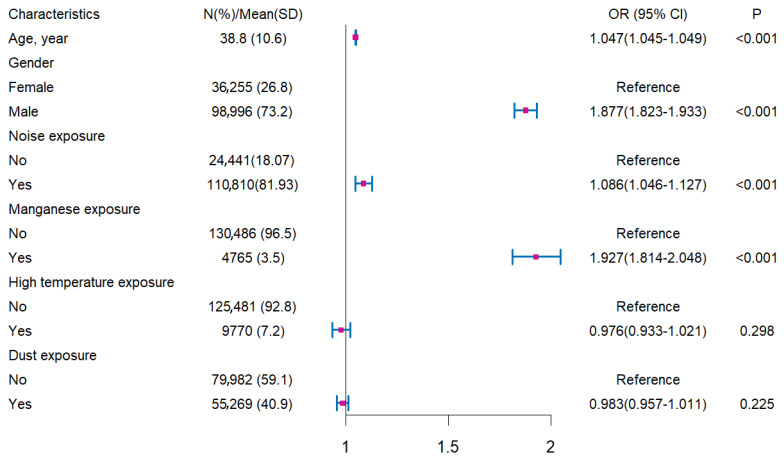
Multivariate logistic regression analysis of occupational noise exposure and age with the risk of HFHL among all participants. Model adjusted for gender, high temperature exposure, dust exposure, and manganese exposure.

Multivariate logistic regression identified age, noise exposure, and manganese exposure as independent risk factors for HFHL, with gender also exerting a modifying effect (Figure 4; Table 4). After adjusting for covariates including high temperature and dust exposure, each one-year increase in age was associated with a 4.7% increase in the odds of HFHL (OR = 1.047, 95% CI: 1.045–1.049, *p* < 0.001). Workers exposed to occupational noise had elevated odds of HFHL compared to their non-exposed peers (OR = 1.079, 95% CI: 1.033–1.126 for males; OR = 1.102, 95% CI: 1.021–1.190 for females), indicating consistent associations across gender. Manganese exposure also markedly increased HFHL odds (male: OR = 1.954; female: OR = 1.698, *p* < 0.001), while high temperature and dust exposures were not statistically significant.

### 3.4. Joint and Interaction Associations Analysis Results

**Figure 5:**
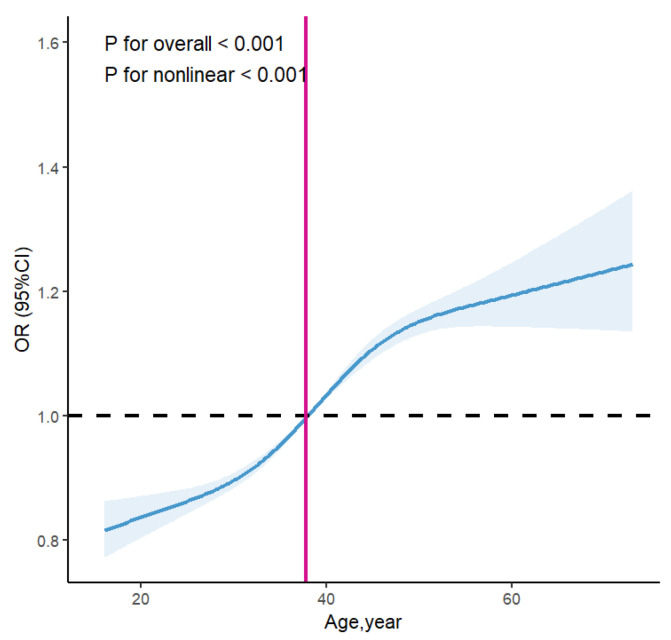
Restricted cubic splines for the associations between age and binaural HFHL of occupational noise-exposed employees. Model adjusted for gender, high temperature exposure, dust exposure, and manganese exposure. The solid blue line represented the multivariate-adjusted risk ratio. The inflection point, which is defined as the point at which the trend changes direction, occurs at approximately 37.769 years of age, as illustrated by the red solid line. And the blue area indicated the 95%Cls derived from restricted cubic spline regression.

RCS regression revealed a significant nonlinear association between age and the risk of HFHL, with an inflection point identified at approximately 37.769 years (*p* for overall trend < 0.001; *p* for nonlinearity < 0.001) (Figure 5). This may reflect a biological turning point where cumulative aging damage begins to exceed compensatory capacity in cochlear structures. Therefore, age was dichotomized at this cutoff for subsequent stratified and interaction analyses with occupational noise exposure.

The joint effect of noise exposure and age on HFHL is shown in Table 5. Participants with both advanced age (>37.769 years) and noise exposure had the highest odds of HFHL (OR = 2.564; 95% CI: 2.456–2.677), compared to the reference group (younger age with no exposure). This finding indicates that older workers exposed to occupational noise are over 2.5 times more likely to experience high-frequency hearing loss, representing a substantial burden with direct implications for occupational safety, productivity, and long-term health costs. Notably, in gender-stratified analyses, males with both exposures had an OR of 2.659 and females had an OR of 2.547, demonstrating that the interaction effect is robust across genders.

Table 6 presents the results of additive and multiplicative interaction analyses. The RERI was 2.075, and the AP was 0.502, indicating that more than 50% of the observed risk in the combined group was due to the interaction between age and noise exposure. The synergy index (SI = 2.967) further confirms a strong supra-additive effect. In terms of multiplicative interaction, the odds ratio for the interaction term was 1.265 (95% CI: 1.176–1.360; *p* < 0.001), indicating a statistically significant interaction effect under a multiplicative framework. This means that the increase in HFHL risk when both exposures are present is greater than the product of the risks from each exposure individually.

### 3.5. Machine Learning Model Analysis Results

**Figure 6:**
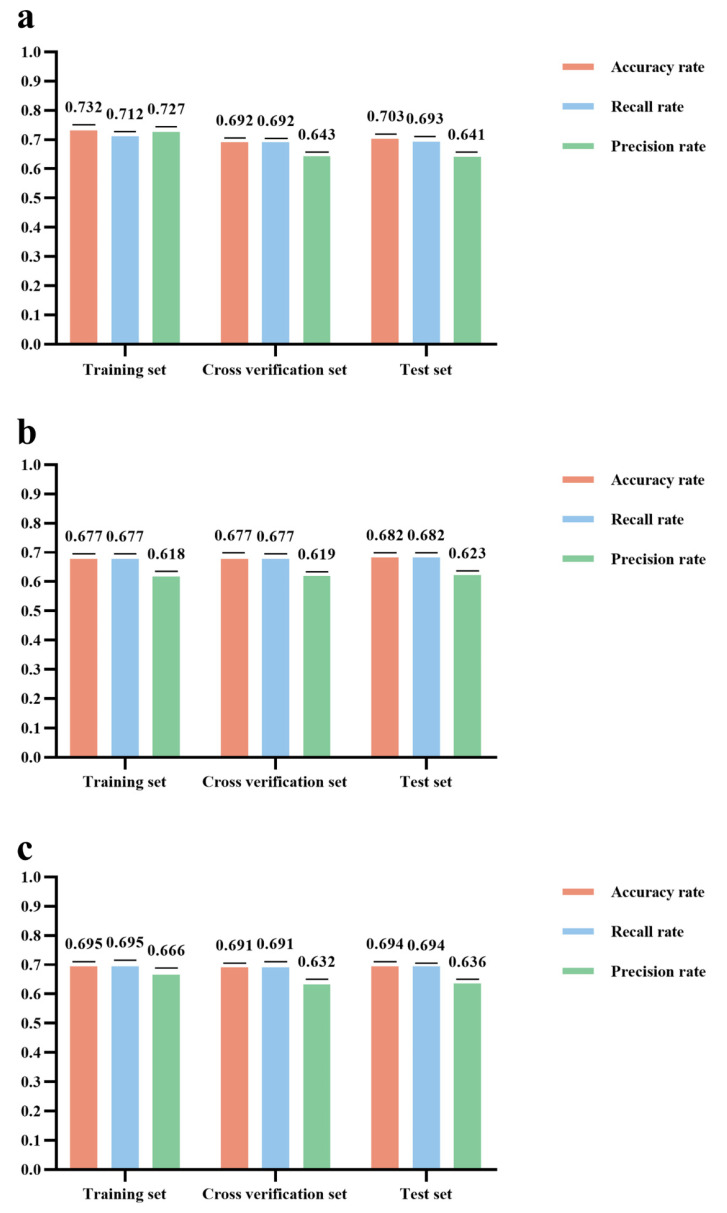
Model evaluation results: (**a**): RFC model evaluation result; (**b**): NBC model evaluation result; (**c**): Catboost model evaluation result.

Figure 6 shows the performance evaluation of the three machine learning models based on accuracy, recall, and precision across the training, cross-validation, and test sets. All models achieved satisfactory performance, with all evaluation metrics exceeding 60.0%. Among them, the RFC model achieved the best overall performance, with an accuracy of 70.3%, a recall of 69.3%, and a precision of 64.1% in the test set. These results indicate that RFC has superior predictive stability and sensitivity in identifying individuals with HFHL.

**Figure 7:**
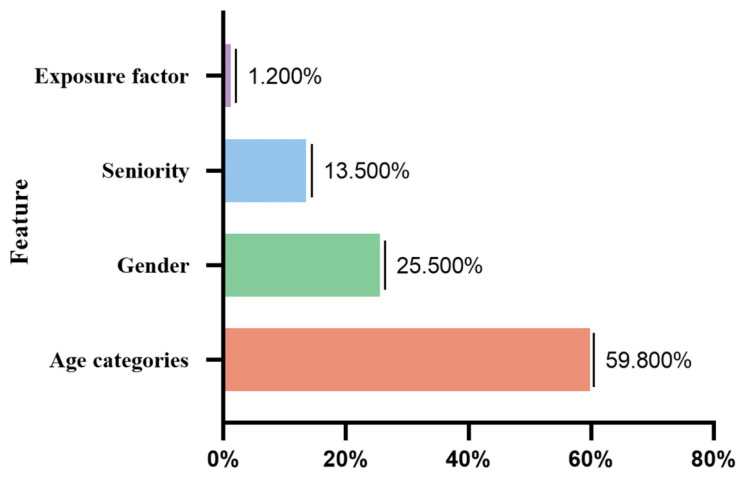
Feature importance of the RFC model.

We further analyzed feature importance using the RFC model (Figure 7). The first four characteristics were age categories (59.8%), gender (25.5%), exposure time seniority (13.5%), and exposure factor (1.2%). The values quantify each feature’s relative contribution to reducing node impurity across all trees in the forest. These findings highlight age, gender, and noise exposure as key predictors, aligning with previous epidemiological evidence.

## 4. Discussion

Globally, occupational noise exposure is on the rise as more countries transition from agrarian to industrial economies [44]. In this context, our study provides important epidemiological insights into the complex interactions between occupational noise exposure and aging. Notably, we identified age, noise exposure, and gender as the most significant predictors of NIHL. Furthermore, a strong interaction was observed between age and noise exposure, suggesting that the combined effects of biological aging and long-term occupational noise exposure may significantly exacerbate auditory decline. These findings expand the current understanding of both ARHL and NIHL and emphasize the need for more targeted occupational health strategies that account for demographic risk factors and their interactions.

Consistent with previous findings [45,46], we observed that both aging and occupational noise exposure emerged as independent yet interacting risk factors for HFHL. In our study, hearing thresholds worsened with advancing age, particularly at higher frequencies, even among individuals not exposed to occupational noise—underscoring aging as a fundamental determinant of auditory decline. The inflection point, identified through restricted cubic spline modeling at approximately 37.8 years, represented a critical biological threshold beyond which cumulative cochlear damage due to intrinsic aging may accelerate rapidly [47,48,49]. However, our results go further by highlighting a supra-additive interaction between noise and age, quantified through multiplicative and additive interaction indices.

Notably, the combined exposure to noise and older age (>37.8 years) was associated with a more than 2.5-fold increase in the odds of HFHL compared to unexposed younger counterparts. This amplification effect supports the “common pathway hypothesis”—a conceptual framework proposing that noise and aging converge on shared molecular targets in the cochlea, including synaptopathy, oxidative stress, mitochondrial dysfunction, and impaired glutamate homeostasis [50,51,52]. Specifically, this interaction may reflect an acceleration of metabolic presbycusis, characterized by a progressive loss of cochlear energy regulation and neural support structures, in contrast to sensory presbycusis, which primarily involves outer hair cell degeneration [53,54,55,56]. While previous studies often consider these pathways separately, our findings reinforce the need to view noise-induced and age-related hearing loss not as isolated entities but as synergistic phenomena that compound cochlear vulnerability over time.

Our findings support growing evidence that gender plays a critical role in susceptibility to NIHL [19,57]. Males exhibited consistently higher median and 95th percentile auditory thresholds, particularly at high frequencies, even after adjusting for age and environmental co-exposures. Multivariate logistic regression further identified being male as an independent risk factor for NIHL. Our stratified analyses reveal that the interaction between noise and age persists across both genders, although subtle gender-specific differences exist. One plausible explanation involves estrogen’s neuroprotective effects in females, which modulate cochlear antioxidant defenses and reduce susceptibility to synaptopathy and outer hair cell damage [58]. Additionally, gender-specific gene expression and auditory neuroanatomy may further contribute to resilience in females [59]. These factors underscore the necessity for gender-specific prevention strategies within the framework of occupational health policy.

Machine learning provides a robust framework for predicting health outcomes from complex, high-dimensional data and has shown increasing utility in occupational hearing loss research. In this study, among the models evaluated, the RFC achieved the best predictive performance, with an accuracy of 70.3%, a recall of 69.3%, and a precision of 64.1% on the test set. These balanced metrics suggest that the RFC model is not only accurate but also sensitive to true HFHL cases, minimizing false negatives. The feature importance analysis corroborated epidemiological findings by identifying age, noise exposure, and gender as the top predictors, reinforcing the validity of our prior multivariate results. Machine learning thus adds confirmatory value by identifying consistent patterns in an independent analytical framework. Future work could integrate deep learning or ensemble hybrid models for enhanced risk stratification and early occupational screening.

A notable aspect of this study is that the considerable proportion of participants exposed to occupational dust—nearly 50% in the noise-exposed group—is consistent with common industrial settings in developing economies. Some studies suggest that airborne dust, especially dust containing metal particulates or silica, may exacerbate auditory damage when combined with noise through enhanced oxidative stress and inflammatory pathways [60,61,62]. Although dust exposure was not statistically significant in our multivariate models, this may reflect residual confounding or misclassification due to reliance on binary self-reports. Nonetheless, the prevalence of dual exposure in our dataset supports calls for integrated hazard control strategies in occupational health, particularly in resource-constrained settings where simultaneous physical and chemical hazards are common. Addressing co-exposures is crucial not only for refining auditory risk assessment but also for guiding multi-factorial intervention strategies.

The present study offers meaningful contributions to occupational health research, particularly for industries characterized by high noise exposure. By identifying age, gender, and noise as independent predictors of HFHL, the findings provide an evidence-based foundation for stratified risk assessment and targeted hearing conservation strategies. For employers, these results emphasize the necessity of tailored interventions for vulnerable subgroups—such as older male workers or those with combined exposures to dust or high temperature—where cumulative auditory risk is elevated. The incorporation of machine learning further strengthens predictive precision, allowing for the early detection of high-risk individuals and more efficient allocation of surveillance resources. For workers, the recognition of multifactorial risks may facilitate improved compliance with personal protective equipment protocols and encourage greater engagement with occupational health education. At a broader level, the findings underscore the inadequacy of noise-only prevention frameworks and advocate for integrated occupational safety programs that account for complex exposure environments. Collectively, the study informs both policy and practice by enhancing our understanding of NIHL determinants and supporting data-driven occupational health management.

This study has several strengths. The large sample size, wide age span, and inclusion of both sexes—particularly a substantial proportion of women exposed to occupational noise—enhance the representativeness and statistical robustness of the findings. Additionally, the use of both traditional regression and machine learning models provides complementary analytical perspectives, strengthening the validity of the conclusions. However, several limitations must be acknowledged. First, the cross-sectional design precludes causal inference between occupational noise exposure and age-related hearing loss. Second, exposure assessment relied on binary classification without incorporating quantitative metrics such as intensity, duration, or cumulative dose, which may obscure exposure–response relationships and introduce misclassification bias. Third, non-occupational noise exposures—such as recreational or environmental sources—were not evaluated, increasing the risk of residual confounding. Moreover, exposure to dust, high temperature, and manganese was primarily self-reported and thus susceptible to recall bias, despite partial record validation. Finally, although the multivariable model accounted for the available covariates, residual confounding due to unmeasured variables—such as socioeconomic status, smoking habits, or exposure to ototoxic agents—may still bias the estimates. These limitations underscore the need for future longitudinal studies incorporating objective exposure quantification and biomarker-based validation to improve causal inference and internal validity.

## 5. Conclusions

In our study, we comprehensively investigated the joint and interacting effects of occupational noise exposure, age, and gender on high-frequency hearing loss. We found that both noise and aging independently increase auditory decline, with a synergistic interaction amplifying the risk. Moreover, male workers exhibited greater susceptibility, highlighting potential biological and occupational disparities. These findings provide new insights into the complex etiology of auditory aging and underscore the necessity for gender-sensitive and age-stratified occupational health interventions to mitigate hearing loss. Promoting early detection and targeted prevention strategies holds important implications for worker well-being and public health policy.

## Figures and Tables

**Table 1:** General characteristics among the study participants (N = 135,251).

Characteristics	Level	All Participants	Non-Noise Exposure	Noise Exposure	*p*
No. participants		135,251	24,441	110,810	
Gender, n (%)	Male	98,996(73.2)	16,886(69.1)	82,110(74.1)	<0.01
	Female	36,255(26.8)	7555(30.9)	28,700(25.9)	
Age, year, mean ± SD		38.8(10.6)	32.3(8.8)	40.3(10.4)	<0.01
Duration of noise exposure, year, mean ± SD		5.3(6.4)	-	6.4(6.5)	<0.01
BHFTA, dB		24.0(11.7)	23.4(8.8)	24.1(12.2)	<0.01
High temperature exposure, n (%)	No	125,481(92.8)	24,410(99.9)	101,071(91.2)	<0.01
	Yes	9770(7.2)	31(0.1)	9739(8.8)	
Dust exposure, n (%)	No	79,982(59.1)	24,329(99.5)	55,653(50.2)	<0.01
	Yes	55,269(40.9)	112(0.5)	55,157(49.8)	
	Yes	6328(4.7)	42(0.2)	6286(5.7)	
Manganese exposure, n (%)	No	130,486(96.5)	24,398(99.8)	106,088(95.7)	<0.01
	Yes	4765(3.5)	43(0.2)	4722(4.3)	

Abbreviations: SD, standard device; BHFTA, binaural high-frequency threshold average.

**Table 2:** Age and gender distribution of employees in this study.

	Noise-Exposed and Control Groups (n = Number of Participants)	
Age Categories and Gender (Males (M) and Females (W))	Noise Group	Control Group
N_16–76years_ = 135,251	N_Noise group_ = 110,810	N_Control group_ = 24,441
16 to 30 years	N_16–30years_ = 22,728	N_16–30years_ = 11,998
M	19,399	8857
F	3329	3141
31 to 40 years	N_31–40years_ = 33,905	N_31–40years_ = 8073
M	24,806	5179
F	9099	2894
41 to 50 years	N_41–50years_ = 33,109	N_41–50years_ = 3411
M	21,134	2016
F	11,975	1395
51 to 60 years	N_51–60years_ = 19,141	N_51–60years_ = 915
M	15,166	800
F	3975	115
61 to 76 years	N_61–76years_ = 1927	N_61–76years_ = 44
M	1605	34
F	322	10

**Table 3:** Median and 95th percentile values of hearing thresholds for the control group categorized by gender.

	Control Group: Male (n = 16,891) Female (n = 7556)					
	Median Values for Thresholds (dB) Per Frequency for the Control Group					
	0.5 kHz	1 kHz	2 kHz	3 kHz	4 kHz	6 kHz
Males	22.5	21.0	20.0	20.5	22.5	22.5
Females	23.0	22.5	22.0	21.0	21.5	23.0
	95th percentile values for thresholds (dB) per frequency for the control group					
	0.5 kHz	1 kHz	2 kHz	3 kHz	4 kHz	6 kHz
Males	26.5	25.5	26.5	38.0	48.0	50.0
Females	28.0	25.5	26.5	31.0	33.5	36.5

**Table 4:** Logistic regression analysis of occupational noise exposure and age with the risk of binaural HFHL among males and females.

Characteristics		Male		Famale	
		OR (95% Cl)	*p*	OR (95% Cl)	*p*
Age		1.047(1.045–1.049)	<0.001	1.049(1.044–1.053)	<0.001
Noise exposure	No	Reference		Reference	
	Yes	1.079(1.033–1.126)	0.001	1.102(1.021–1.190)	0.013
Manganese exposure	No	Reference		Reference	
	Yes	1.954(1.831–2.084)	<0.001	1.698(1.404–2.053)	<0.001
High temperature exposure	No	Reference		Reference	
	Yes	0.968(0.922–1.017)	0.197	1.035(0.913–1.173)	0.592
Dust exposure	No	Reference		Reference	
	Yes	0.989(0.958–1.02)	0.466	0.966(0.911–1.024)	0.24

NE: Noise exposure. Data are expressed as odds ratios (ORs) and 95% CI. Model adjusted for high temperature exposure, dust exposure, and manganese exposure.

**Table 5:** Joint association of occupational noise exposure and age with the risk of binaural HFHL among participants.

Characteristics			All		Male		Famale	
			OR (95% CI)	*p*	OR (95% CI)	*p*	OR (95% CI)	*p*
Age	Low level	Non-NE	Reference		Reference		Reference	
		NE	1.064(1.017–1.113)	0.007	1.028(0.977–1.081)	0.291	1.151(1.039–1.274)	0.007
	High level	Non-NE	1.906(1.783–2.038)	<0.001	1.998(1.847–2.160)	<0.001	2.028(1.782–2.308)	<0.001
		NE	2.564(2.456–2.677)	<0.001	2.659(2.659–2.933)	<0.001	2.547(2.327–2.787)	<0.001

NE: Noise exposure. Data are expressed as odds ratios (ORs) and the 95% CI. Model adjusted for gender, high temperature exposure, dust exposure, and manganese exposure.

**Table 6:** Interactive analysis of occupational noise exposure and age with the risk of binaural HFHL among participants.

Additive Interactive				Multiplicative Interactive	
Measure	Estimate	Lower	Upper	OR (95% CI)	*p*
RERI	2.075	1.803	2.347	1.265(1.176–1.36)	<0.001
AP	0.502	0.484	0.521		
SI	2.967	2.818	3.124		

NE: Noise exposure. Data are expressed as odds ratios (ORs) and the 95% CI. Model adjusted for gender, high temperature exposure, dust exposure, and manganese exposure.

## Data Availability

The data presented in this study are available from the corresponding author upon request due to ethical restrictions. The data include sensitive personal health information that cannot be publicly shared in order to protect participant confidentiality, in accordance with the consent agreements and institutional ethical guidelines.

## Abbreviations

NIHL	Noise-induced hearing loss
ARHL	Age-related hearing loss
HFHL	High-frequency hearing loss
NID	Noise-induced deafness
PTA	Pure-tone audiometry
BHFTA	Binaural high-frequency threshold average
RCS	Restricted cubic spline
RFC	Random forest classification model
RERI	Relative Excess Risk due to Interaction
AP	Attributable Proportion
SI	Synergy Index
NBC	Naive Bayesian classification model

## References

[1] Stucken E.Z., Hong R.S. (2024). Noise-induced hearing loss: An occupational medicine perspective. Curr. Opin. Otolaryngol. Head Neck Surg..

[2] Ryan A.F., Kujawa S.G., Hammill T., Le Prell C., Kil J. (2016). Temporary and Permanent Noise-induced Threshold Shifts: A Review of Basic and Clinical Observations. Otol. Neurotol..

[3] Nelson D.I., RNelson Y., Concha-Barrientos M., Fingerhut M. (2005). The global burden of occupational noise-induced hearing loss. Am. J. Ind. Med..

[4] Śliwińska-Kowalska M., Zaborowski K. (2017). WHO Environmental Noise Guidelines for the European Region: A Systematic Review on Environmental Noise and Permanent Hearing Loss and Tinnitus. Int. J. Environ. Res. Public Health.

[5] Chadha S., Kamenov K., Cieza A. (2021). The world report on hearing, 2021. Bull. World Health Organ..

[6] Zhou J., Shi Z., Zhou L., Hu Y., Zhang M. (2020). Occupational noise-induced hearing loss in China: A systematic review and meta-analysis. BMJ Open.

[7] Hu Y., Wang J.X., Zhang M.B. (2021). Research progress on non-steady state noise-induced hearing loss. Zhonghua Lao Dong Wei Sheng Zhi Ye Bing Za Zhi.

[8] Kiely K.M., Gopinath B., Mitchell P., Luszcz M., Anstey K.J. (2012). Cognitive, Health, and Sociodemographic Predictors of Longitudinal Decline in Hearing Acuity Among Older Adults. J. Gerontol. A Biol. Sci. Med. Sci..

[9] Mick P., Kawachi I., Lin F.R. (2014). The Association between Hearing Loss and Social Isolation in Older Adults. Otolaryngol. Head Neck Surg..

[10] Arjmandi M.K., Neils-Strunjas J., Nemati S., Fridriksson J., Newman-Norlund S., Newman-Norlund R., Bonilha L. (2024). Age-Related Hearing Loss, Cognitive Decline, and Social Interaction: Testing a Framework. J. Speech Lang. Hear. Res..

[11] McMahon C.M., Gopinath B., Schneider J., Reath J., Hickson L., Leeder S.R., Mitchell P., Cowan R. (2013). The Need for Improved Detection and Management of Adult-Onset Hearing Loss in Australia. Int. J. Otolaryngol..

[12] Lin F.R., Niparko J.K., Ferrucci L. Hearing Loss Prevalence in the United States. https://jamanetwork.com/journals/jamainternalmedicine/fullarticle/1106004.

[13] (2013). Acoustics—Estimation of Noise-Induced Hearing Loss.

[14] Keithley E.M. (2020). Pathology and mechanisms of cochlear aging. J. Neurosci. Res..

[15] Hawkins J.E. (1973). Comparative otopathology: Aging, noise, and ototoxic drugs. Adv. Otorhinolaryngol..

[16] Gates G.A. (2006). The effect of noise on cochlear aging. Ear Hear..

[17] Viana L.M., Neils-Strunjas J., Nemati S., Fridriksson J., Newman-Norlund S., Newman-Norlund R., Bonilha L. (2015). Cochlear neuropathy in human presbycusis: Confocal analysis of hidden hearing loss in post-mortem tissue. Hear. Res..

[18] Möhrle D., Ni K., Varakina K., Bing D., Lee S.C., Zimmermann U., Knipper M., Rüttiger L. (2016). Loss of auditory sensitivity from inner hair cell synaptopathy can be centrally compensated in the young but not old brain. Neurobiol. Aging.

[19] Wu P.Z., Liberman L.D., Bennett K., de Gruttola V., O’Malley J.T., Liberman M.C. (2019). Primary Neural Degeneration in the Human Cochlea: Evidence for Hidden Hearing Loss in the Aging Ear. Neuroscience.

[20] Wang Q., Wang X., Yang L., Han K., Huang Z., Wu H. (2021). Sex differences in noise-induced hearing loss: A cross-sectional study in China. Biol. Sex Differ..

[21] Charitidi K., Meltser I., Tahera Y., Canlon B. (2009). Functional responses of estrogen receptors in the male and female auditory system. Hear. Res..

[22] Reavis K.M., Bisgaard N., Canlon B., Dubno J.R., Frisina R.D., Hertzano R., Humes L.E., Mick P., Phillips N.A., Pichora-Fuller M.K. (2023). Sex-Linked Biology and Gender-Related Research Is Essential to Advancing Hearing Health. Ear Hear..

[23] Rouhbakhsh N.A., Al-Mnaseer R.A.D., Mohamadkhani G. (2025). Occupational Noise-Induced Hearing Loss in Cotton mill workers in Baghdad, Iraq. J. Paramed. Sci. Rehabil..

[24] Nieman C.L., Suen J.J., Dean L.T., Chandran A. (2022). Foundational Approaches to Advancing Hearing Health Equity: A Primer in Social Epidemiology. Ear Hear..

[25] Meira T.C., Santana V.S., Ferrite S. (2015). Gender and other factors associated with the use of hearing protection devices at work. Rev. Saúde Pública.

[26] (2008). Emisson Standard for Industrial Enterprises Noise at Boundary.

[27] (2018). Acoustics—Audiometric Test Methods.

[28] (2014). Diagnosis of Occupational Noise-Induced Deafness.

[29] Wang D., Xiao Y., Feng X., Wang B., Li W., He M., Zhang X., Yuan J., Yi G., Chen Z. (2021). Association of occupational noise exposure, bilateral hearing loss with atherosclerotic cardiovascular disease risk in Chinese adults. Int. J. Hyg. Environ. Health.

[30] (2007). Measurement of Physical Agents in Workplace Part 8: Noise.

[31] (2022). Occupational Noise Exposure.

[32] Imani M., Beikmohammadi A., Arabnia H.R. (2025). Comprehensive Analysis of Random Forest and XGBoost Performance with SMOTE, ADASYN, and GNUS Under Varying Imbalance Levels. Technologies.

[33] Ali J. (2012). Random Forests and Decision Trees.Pdf. Random For. Decis. Trees.

[34] Sethi J.K., Mittal M. (2022). Efficient weighted naive bayes classifiers to predict air quality index. Earth Sci. Inform..

[35] (2021). Variable selection for Naïve Bayes classification. Comput. Oper. Res..

[36] Hancock J.T., Khoshgoftaar T.M. (2020). CatBoost for big data: An interdisciplinary review. J. Big Data.

[37] Zamzam Y.F., Saragih T.H., Herteno R., Muliadi, Nugrahadi D.T., Huynh P.-H. (2024). Comparison of CatBoost and Random Forest Methods for Lung Cancer Classification using Hyperparameter Tuning Bayesian Optimization-based. J. Electron. Electromed. Eng. Med. Inform..

[38] Tanha J., Abdi Y., Samadi N., Razzaghi N., Asadpour M. (2020). Boosting methods for multi-class imbalanced data classification: An experimental review. J. Big Data.

[39] Song Y., Yin Z., Zhang C., Hao S., Li H., Wang S., Yang X., Li Q., Zhuang D., Zhang X. (2022). Random forest classifier improving phenylketonuria screening performance in two Chinese populations. Front. Mol. Biosci..

[40] Gote P.M., Kumar P., Kumar H., Verma P., Jiet M.M. (2025). Integrating Machine Learning Algorithms: A Hybrid Model for Lung Cancer Outcome Improvement. Appl. Sci..

[41] Stefano A., Bini F., Giovagnoli E., Dimarco M., Lauciello N., Narbonese D., Pasini G., Marinozzi F., Russo G., D’Angelo I. (2025). Comparative Evaluation of Machine Learning-Based Radiomics and Deep Learning for Breast Lesion Classification in Mammography. Diagnostics.

[42] Yang T., Chen H.J., Zhang C.Y., He D., Yuan W. (2024). Association of blood heavy metal concentrations with hearing loss: A systematic review and meta-analysis. Public Health.

[43] Muthaiah V.P.K., Chen G.D., Ding D., Salvi R., Roth J.A. (2016). Effect of manganese and manganese plus noise on auditory function and cochlear structures. NeuroToxicology.

[44] Chen Z., Li W., Zhang H., Huang X., Tao Y., Lang K., Zhang M., Chen W., Wang D. (2024). Association of noise exposure, plasma microRNAs with metabolic syndrome and its components among Chinese adults. Sci. Total Environ..

[45] Lie A., Skogstad M., Johannessen H.A., Tynes T., Mehlum I.S., Nordby K.C., Engdahl B., Tambs K. (2016). Occupational noise exposure and hearing: A systematic review. Int. Arch. Occup. Environ. Health.

[46] Gopinath B., McMahon C., Tang D., Burlutsky G., Mitchell P. (2021). Workplace noise exposure and the prevalence and 10-year incidence of age-related hearing loss. PLoS ONE.

[47] Persic D., Thomas M.E., Pelekanos V., Ryugo D.K., Takesian A.E., Krumbholz K., Pyott S.J. (2020). Regulation of auditory plasticity during critical periods and following hearing loss. Hear. Res..

[48] Fu X., Sun X., Zhang L., Jin Y., Chai R., Yang L., Zhang A., Liu X., Bai X., Li J. (2018). Tuberous sclerosis complex–mediated mTORC1 overactivation promotes age-related hearing loss. J. Clin. Investig..

[49] Pinheiro B.P., Vona B., Löwenheim H., Rüttiger L., Knipper M., Adel Y. (2021). Age-related hearing loss pertaining to potassium ion channels in the cochlea and auditory pathway. Pflug. Arch. Eur. J. Physiol..

[50] Tan W.J.T., Song L. (2023). Role of mitochondrial dysfunction and oxidative stress in sensorineural hearing loss. Hear. Res..

[51] Wan H., Wang W., Liu J., Zhang Y., Yang B., Hua R., Chen H., Chen S., Hua Q. (2023). Cochlear metabolomics, highlighting novel insights of purine metabolic alterations in age-related hearing loss. Hear. Res..

[52] Yang Z.-J., Zhao C.-L., Liang W.-Q., Chen Z.-R., Du Z.-D., Gong S.-S. (2024). ROS-induced oxidative stress and mitochondrial dysfunction: A possible mechanism responsible for noise-induced ribbon synaptic damage. Am. J. Transl. Res..

[53] Zhao X., Shen T., Cao S., Liu Z., Pang W., Li M., Liu J., Li W., Wu Y., Liu C. (2025). Presbycusis: Pathology, Signal Pathways, and Therapeutic Strategy. Adv. Sci..

[54] Liu H., Giffen K.P., Chen L., Henderson H.J., Cao T.A., Kozeny G.A., Beisel K.W., Li Y., He D.Z. (2022). Molecular and cytological profiling of biological aging of mouse cochlear inner and outer hair cells. Cell Rep..

[55] Fuentes-Santamaría V., Alvarado J.C., Mellado S., Melgar-Rojas P., Gabaldón-Ull M.C., Cabanes-Sanchis J.J., Juiz J.M. (2022). Age-Related Inflammation and Oxidative Stress in the Cochlea Are Exacerbated by Long-Term, Short-Duration Noise Stimulation. Front. Aging Neurosci..

[56] Zou S., Liu Y., Xu B., Li J., He Z. (2025). Age-related hearing loss: The complex interaction of carbohydrate metabolism in auditory and cognitive dysfunction during aging. Ageing Neur. Dis..

[57] Villavisanis D.F., Berson E.R., Lauer A.M., Cosetti M.K., Schrode K.M. (2020). Sex-Based Differences in Hearing Loss: Perspectives from Non-Clinical Research to Clinical Outcomess. Otol. Neurotol..

[58] Lien K.-H., Yang C.-H. (2021). Sex Differences in the Triad of Acquired Sensorineural Hearing Loss. Int. J. Mol. Sci..

[59] Nolan L.S. (2020). Age-related hearing loss: Why we need to think about sex as a biological variable. J. Neurosci. Res..

[60] Mirmohammadi S., Khanjani N., Nazarkhani F., Abediankenari S., Yazdani J., Tilaki R.A.D. (2020). The effect of noise and dust exposure on oxidative stress among livestock and poultry feed industry workers. Toxicol. Ind. Health.

[61] Duan D., Leng P., Li X., Mao G., Wang A., Zhang D. (2023). Characteristics and occupational risk assessment of occupational silica-dust and noise exposure in ferrous metal foundries in Ningbo, China. Front. Public Health.

[62] Safinejad M., Azari M.R., Zendehdel R., Rafieepour A., Khodakarim S., Khodarahmi B. (2019). Occupational and biological monitoring of workers exposed to airborne dust in Gol-e-Gohar Iron Ore mine: A Case-Control Study. Iran. Occup. Health.

